# Rooted in routine: Fostering higher order vegetable‐shopping habits using a randomised simple planning intervention

**DOI:** 10.1111/aphw.12649

**Published:** 2025-01-30

**Authors:** Kimberly R. More, Curt More, Natasha Harris, L. Alison Phillips

**Affiliations:** ^1^ Department for Health University of Bath Bath UK; ^2^ Department of Psychology University of Dundee Dundee UK; ^3^ Department of Psychology University of Bath Bath UK; ^4^ Department of Psychology Iowa State University Ames Iowa USA

**Keywords:** behavioural automaticity, behavioural maintenance, habit formation, health behaviour intervention, planning intervention, vegetable intake

## Abstract

A healthy diet is a protective factor against a host of negative health outcomes. To maintain such a diet necessitates the consumption of at least 240 g of vegetables per day. However, most of the population fails to meet this threshold. Utilising a randomised controlled trial, the present study tested the effectiveness of a one‐off higher order habit intervention aimed at shopping for a variety of vegetables and the mechanisms that may support such habit development. Specifically, participants (*N* = 198; 54.5% female; 20 to 74 years of age) were allocated to the intervention or control group to explore (1) how effective an action‐ and coping‐planning intervention is at targeting the formation of vegetable‐shopping higher order habits and (2) whether healthy‐eater identity, intrinsic motivation and self‐efficacy were mechanisms of action. Follow‐up measures of habit, the mechanisms of action and behaviour were taken post‐intervention, weekly for 4 weeks and at 3‐ and 6‐month follow‐ups. The intervention led to stronger higher order habit formation after 6 months and that it was particularly effective for those with low baseline higher order habits for vegetable shopping. These findings demonstrate that a simple, one‐off, intervention can lead to long‐lasting change in higher order habits within the nutrition domain.

## INTRODUCTION

Nutrition is intertwined with health as a healthy diet is a key protector against malnutrition and noncommunicable diseases, such as type 2 diabetes, heart disease, stroke and certain types of cancer (Wang et al., 2021; World Health Organization, 2019). For adults, part of a healthy diet involves consuming at least three servings, or 240 g, of vegetables per day (Kalmpourtzidou et al., 2020; Wang et al., 2021; World Health Organization, 2003). However, the worldwide average daily vegetable intake is approximately two servings, 186 g, which results from 88% of countries having an average daily intake below the recommended minimum (Kalmpourtzidou et al., 2020). In addition to total vegetable consumption, it is also important to incorporate a variety of vegetables to promote optimal health as such variety has been linked to decreased low‐grade inflammation, greater overall daily consumption and decreased incidence of certain types of cancer and type 2 diabetes (e.g. Almeida‐de‐Souza et al., 2018; Büchner et al., 2010; Cooper et al., 2012; Meengs et al., 2012). Higher order habit interventions are one means that may help individuals to initiate and maintain health behaviours that require varied execution to achieve optimal health benefits, such as nutrition‐related behaviours (Phillips et al., 2019).

Habitual behaviours are those that are instigated (deciding to do) and/or executed (doing) automatically (Gardner et al., 2016), with instigation habits being more strongly related to behavioural frequency than execution habits (Phillips et al., 2016). Both instigation and execution habits form by regularly pairing a specific cue with a specific rewarding behaviour (e.g. when I eat breakfast, I will eat a grapefruit; Phillips et al., 2016; Wood & Neal, 2007). Once a habit is formed, exposure to the associated cue triggers the recall of the behavioural sequence stored in procedural memory (Wood & Neal, 2007; Wood & Rünger, 2016). Habits to engage in positive health behaviours are advantageous as they trigger the behaviour automatically and are less vulnerable to fluctuations to other psychological processes like motivation and cognitive capacity compared with non‐habitual behaviours (Lally & Gardner, 2013). However, habits are traditionally thought of in relation to a single behaviour or a chunked sequence of specific actions triggered by a conditioned cue (e.g. Wood & Neal, 2007), which undermines the behaviour variability needed for the optimisation of many health behaviours (e.g. eating a variety of produce). To address this limitation, Phillips and colleagues first proposed and tested the theoretical construct of higher order habits in 2019.

Higher order habits allow for behavioural variety in response to a conditioned cue (Phillips et al., 2019). For example, an individual may consistently serve themselves vegetables to have with their dinner, but the vegetables served can vary (e.g. asparagus, broccoli, carrots). Theoretically, a higher order habit is an instigation habit as it involves habitually engaging in a behaviour, in response to a conditioned cue, which persists with either no execution habit or a variety of execution habits (Gardner et al., 2016; Phillips et al., 2019; Phillips & Gardner, 2016). In their seminal study, Phillips et al. (2019) conducted a randomised controlled trial to examine the feasibility and utility of the higher order habits construct. Undergraduate students who frequented a university dining hall were randomised into an intervention or control group. Both groups received the behavioural goal of filling half their dinner plates with fruits and vegetables, and the intervention group also created action and coping plans for doing so. Participants in both groups were successful at consuming a variety of produce. However, participants in the intervention group were more successful at, and formed stronger higher order habits for, consuming fruit and vegetable over the course of 4 weeks.

Kilb and Labudek (2022) extended upon the research on higher order habits in the nutrition domain using an online intervention that focused exclusively on vegetables in a more real‐world context as participation did not require access to pre‐prepared foods in a dining hall. All participants received the goal of filling half their dinner plates with vegetables and received an action and coping planning intervention. Higher order habit strength for filling half of dinner plates with vegetables increased over the 56‐day measurement period, especially for those who performed the goal behaviour more regularly and those who enjoyed consuming vegetables. Together these studies demonstrate that higher order habit interventions have the potential to impact the regular consumption of a variety of produce. However, this preliminary research is not without limitations and experts in the domain of health‐related habits have called for more evaluative research on the utility of higher order habit interventions (Gardner et al., 2023).

Specifically, these proof‐of‐concept studies assessed habit over the course of 1 or 2 months. These shorter, prospective designs are unable to give insight into whether participants enter the maintenance phase of behaviour change, which has been traditionally defined as 6 months post‐initiation within the transtheoretical model (Prochaska & Velicer, 1997), but 3 months has also been used as a less conservative benchmark (Phillips et al., 2016). Moreover, although these studies provide an initial conceptual test of higher order habit development in the nutrition domain, both obscure the true complexity of eating behaviours. Nutrition behaviours and related habits are complex and require multiple steps, such as meal planning, grocery shopping, meal preparation, dishing out food and consumption (Phillips & Mullan, 2022; Saunders & More, 2024). Phillips et al. (2019) control for the initial steps of planning, shopping and preparation by having participants consume produce from a dining hall where prepared foods are served. Conversely, Kilb and Labudek (2022) do not address these initial nutrition behaviours in their intervention. Thus, these interventions may only be advantageous for those who already have access to produce—specifically, vegetables—within their eating environments and who have food preparation knowledge. Finally, and more broadly, in their exploration of remaining questions in the habit literature, Gardner et al. (2023) proposed that the distinction between higher and lower habits is difficult to operationalise. Specifically, they theorise that these terms are relative rather than absolute, as their definitions can shift depending on context. For instance, the habitual act of buying vegetables may occur within the broader context of grocery shopping, which may also be habitual. This suggests that this would make the act of vegetable shopping a lower order habit relative to the act of grocery shopping. However, this context‐dependent distinction is not particularly significant, as the presence of a habit within an existing routine does not alter the classification of a habitually instigated behaviour having execution variety (higher order) or not (lower order). Thus, if a person habitually grocery shops for a variety of vegetables within the context of also habitually shopping for other items, both behaviours would still be considered higher order, as the definition does not change based on the broader context.

Although limitations to the current higher order habit research have been raised, it is important to note that the empirical evidence (i.e. Kilb & Labudek, 2022; Phillips et al., 2019) has consistently demonstrated that higher order nutrition habits can be formed from the outset much like lower order habits. That is, higher order habits can form with behavioural repetition, regardless of variation in execution. However, the mechanisms underpinning higher order habit formation remain relatively underexplored. Phillips et al. (2019) reported that intrinsic motivation for consuming produce does not serve as a mechanism for higher order habit formation. In contrast, Kilb and Labudek (2022) identified repeated behavioural performance, intrinsic reward value and context stability as key mechanisms contributing to higher order habit formation. In the present study, we examined mechanisms of action for higher order habit formation within the context of the habit formation framework (Gardner & Lally, 2018; Lally & Gardner, 2013). This framework outlines four stages of habit formation, each characterised by distinct mechanisms of action: (1) decision‐making, (2) self‐regulation, (3) behavioural repetition and (4) formation of cue‐behaviour associations. This research centres on four key mechanisms influencing (in)action: self‐efficacy, identity, intrinsic motivation and perceived barriers to behaviour, which span the first three phases of the habit formation framework, though this list is not exhaustive.

For the first phase of the habit formation framework (i.e. decision‐making), we examined self‐efficacy as a mechanism of action (e.g. Lally & Gardner, 2013). Self‐efficacy refers to an individual's belief in their capability to successfully engage in a specific behaviour (Bandura, 1994). Specifically, self‐efficacy predicts behavioural intentions for individuals who have not yet reached the maintenance phase of behaviour change (More & Phillips, 2022) and self‐efficacy has been specifically linked to habit formation (Stojanovic et al., 2021).

For the second phase of the habit formation framework (i.e. self‐regulation), we examined identity as a mechanism of action (e.g. Lally & Gardner, 2013). Identity is the notion that a behaviour is an important part of one's self‐concept (Stets & Burke, 2000). Identity theory posits that adopting a behaviour‐related identity integrates performance standards into one's self‐concept, facilitating behavioural regulation. Acting in ways consistent with one's behavioural identity generates positive affect, while identity‐inconsistent actions produce negative affect, thereby encouraging behavioural realignment (Higgins, 1987; Stets & Burke, 2000). Identity has been linked to behavioural engagement, including in the nutrition domain (e.g. Caldwell et al., 2024) and has been shown to be a necessary but insufficient condition of habit (More & Phillips, 2021).

For the third phase of the habit formation framework (i.e. behavioural repetition), we examined both intrinsic motivation and perceived barriers to engagement (e.g. Lally & Gardner, 2013). Intrinsic motivation is the most autonomous form of motivation as proposed by self‐determination theory and refers to an individual engaging in behaviours because they find them enjoyable (Ryan & Deci, 2000). Intrinsic motivation is linked to frequency of behavioural engagement (e.g. Michaelidou et al., 2012). In addition, although Phillips et al. (2019) found no impact of intrinsic motivation on higher order habit formation, previous research has found that behavioural frequency is a stronger predictor of habit for those who are autonomously motivated (Gardner & Lally, 2013). Finally, perceived barriers are beliefs about obstacles that could get in the way of behaviour change and engagement. Perceived barriers are associated with disengagement with many health behaviours, including eating healthfully, which precludes habit formation (Ross & Melzer, 2016).

**FIGURE 1:**
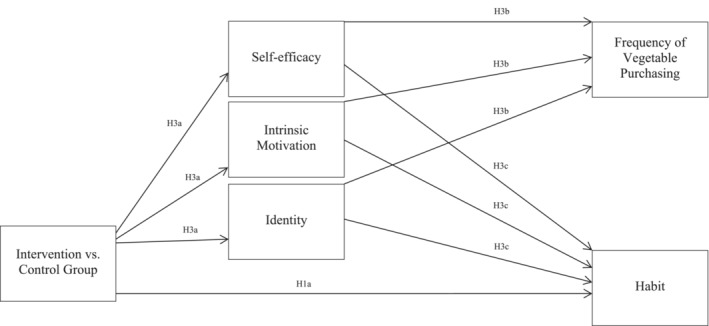
Proposed mechanism of action.

**FIGURE 2:**
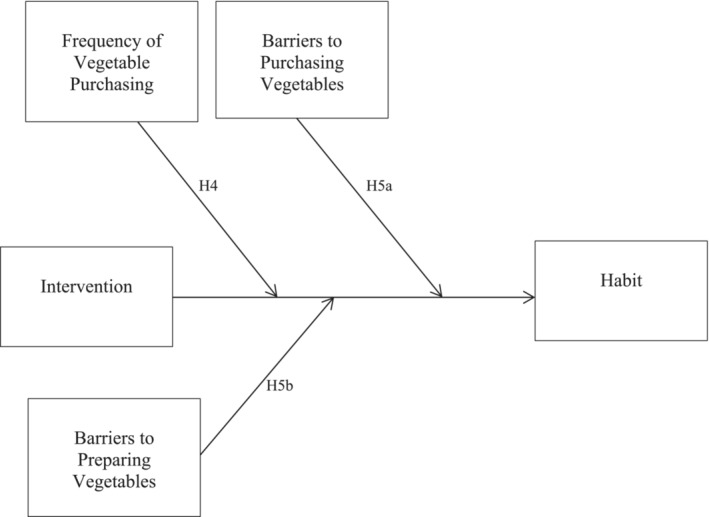
Moderators of habit formation within the intervention group.

Building on past studies, the present study tested the utility of a higher order habit intervention for the initial step of most nutrition behaviours—shopping—and examined potential mechanisms of higher order habit formation. This study set out to examine (1) how effective an ‘action‐planning and coping‐planning’ intervention is at helping individuals form vegetable‐shopping habits (i.e. a ‘higher order habit’) compared with a control group and (2) whether healthy‐eater identity, intrinsic motivation and self‐efficacy were mechanisms of action for the formation of a higher order vegetable‐shopping habit. To assess these research questions, a two‐group randomised controlled trial was conducted with both groups receiving education on vegetable preparation and the goal to increase access to vegetables within the home environment to have with dinner. In addition to this, the intervention group received an action‐ and coping‐planning intervention alongside mental imagery. The planning intervention targeted pairing chosen cues such as shopping for dinner ingredients with the target behaviour of purchasing at least two types of vegetables to have with dinner. The planning intervention served to facilitate higher order habit formation, which should theoretically help participants achieve maintenance of the goal behaviour (e.g. Rhodes, 2021). Five primary hypotheses were assessed (Figures 1 and 2):

Hypothesis 1a was that participants given an action‐ and coping‐planning intervention would form stronger higher order habits for vegetable shopping in comparison with control participants over the 4‐week period and 3‐ and 6‐month follow‐ups (i.e. at each time point excluding baseline). In an exploratory analysis, habit scores at baseline were entered, first as a covariate and second as a moderator, to determine if the intervention was more effective for intervention participants versus control participants who did not have a habit at intake given the range in habit scores at baseline.

Moreover, in line with Phillips et al. (2019), we expected no significant group differences on number of vegetables purchased per week and the variety of vegetables purchased across weeks. Specifically, (H1b) participants in both the intervention and control groups were expected to purchase two or more types of vegetables per week and it was predicted that there would be no significant difference between the two groups. Additionally, (H1c) participants in both the intervention and control groups were expected to vary in the types of vegetables they purchased on a week‐to‐week basis and it was predicted that there would be no significant difference between the two groups. H1b–c exclude the possibility that only a lower order execution habit has been formed.

For hypothesis 2, it was predicted that participants given an action‐ and coping‐planning intervention would have higher self‐efficacy, in comparison with control participants, immediately post‐intervention. Moreover, it was expected that there would be no differences between the two groups on healthy‐eater identity, habit, or intrinsic motivation for purchasing or eating vegetables immediately post intervention.

Hypothesis 3a was that self‐efficacy, intrinsic motivation for consuming and shopping for vegetables, and healthy‐eater identity would be mechanisms of action of the intervention. Specifically, self‐efficacy, intrinsic motivation for consuming and shopping for vegetables, and healthy‐eater identity would increase more strongly over time for the intervention group in comparison with the control group. Further, H3b reported that the mechanisms of action would predict the frequency of grocery shopping trips where participants purchased at least two types of vegetables and H3c stated that the mechanisms of action would predict habit formation.

For hypothesis 4, it was predicted that those who engaged in more grocery shopping trips where vegetables were purchased, post‐intervention, within the intervention group would have a higher level of habit formation for shopping for vegetables. That is, those that had more opportunities to practice pairing their target behaviour with their chosen cue would be more successful.

Finally, for hypothesis 5a, it was predicted that perceived barriers would moderate the impact of the intervention insofar that intervention group participants who perceived more barriers to purchasing vegetables would have lower levels of habit formation for vegetable shopping and that (H5b) the intervention group participants who perceived more barriers to preparing vegetables would have lower levels of habit formation for vegetable shopping.

## METHOD

This study was approved by the School of Humanities, Social Sciences, and Law Research Ethics Committee at the University of (The University of Dundee). The data collection plan, hypotheses and planned analyses were all preregistered on the Open Science Framework prior to data collection with regard to the first 3 months of the study (https://osf.io/zgckf). Moreover, an additional 6‐month follow‐up timepoint was preregistered prior to data collection (https://osf.io/2rcnw). Reporting is in accordance with CONSORT 2010 guidelines (insert OSF link with updated page numbers during copy‐editing).

### Participants

Participants (*N* = 203) were recruited through the online participant recruitment service Prolific and were paid in accordance with Prolific's guideline of rates being equal to, or in excess of, £6 per hour at the time of data collection. Inclusion criterion required that participants be at least 18 years of age, reside in the United Kingdom, consume fewer than three servings of vegetables per day and identify as the primary grocery shopper for their household. Age and residency were preselected using Prolific pre‐screeners. Primary household grocery shopper status was preselected using Prolific pre‐screeners and was validated using a pre‐screening questionnaire. Daily vegetable consumption was assessed using the pre‐screening questionnaire.

### Procedure

Participants first completed a brief pre‐screening questionnaire for which they were paid £0.37 upon completion, regardless of their eligibility for the main study. Consent was obtained through an active consent procedure. Eligible participants were given access to the main study for which they were paid £5.63 for the first portion (i.e. baseline and experimental manipulation). Participants signed an informed consent form before answering baseline measures and being randomly assigned to either the intervention or control group. Participants further completed four weekly surveys as well as two follow‐up surveys at 3 and 6 months post‐intervention. For each of these surveys, participants were paid £1.25. Participants had 48 h to complete the weekly and 3‐month follow‐up surveys and 7 days to complete the 6‐month follow‐up timepoint. Participants needed to complete the pre‐screening survey, the baseline survey and the week 1 survey to be eligible for participation in the entirety of the study. Participants who completed all measurement timepoints that occurred within the first 3 months were given a bonus of £10. Participants were given an additional £1 bonus if they completed the 6‐month follow‐up survey (More et al., 2024). Baseline data collection occurred on November 7, 2022, with weekly follow‐up data being collected between 14 November 2022 and 7 December 2022. The 3‐month follow‐up was collected between 6 February 2023 and 7 February 2023. The 6‐month follow‐up was collected between 1 May 2023 and 8 May 2023.

### Measures

#### Pre‐screening measures

To assess primary grocery shopper status, participants were asked ‘Over the next four weeks, will you do most of your own grocery shopping (in person or online)?’ Response options were ‘yes’ or ‘no’. To assess daily vegetable consumption, participants were first given an infographic depicting serving size for various types of common vegetables (e.g. onion, pepper, tomato, lettuce, cucumber, broccoli; see https://osf.io/7zsx9/). Serving sizes were determined based on the National Health Services Rough Guide for produce portion sizes (n.d.). Participants were asked ‘Over the last seven days, on how many days did you eat vegetables?’. Response options ranged from ‘0’ to ‘7’. Participants were further asked ‘On average, on the days when you ate vegetables, how many servings did you eat? (Please see the above infographic for examples).’ Response options ranged from ‘0’ to ‘21 or more average servings per day’. The number of days participants consumed vegetables was multiplied by the number of average servings that they reported consuming per day. Participants were further asked ‘is the number of servings of vegetables that you ate over the previous seven days representative of a typical week of eating for you?’. Response options were ‘yes’ and ‘no’.

#### Baseline measures

##### Pre‐intervention measures

Prior to being randomly allocated to the control or intervention group (randomisation was done at a 1:1 ratio, without replacement, using Gorilla survey software), participants reported on their perceived barriers to both buying and preparing vegetables using the items provided by Landry et al. (2020). Three items were assessed regarding barriers to buying vegetables (e.g. ‘Vegetables were too expensive’; *α* = .453), and four items were used to assess perceived barriers to preparing and cooking vegetables (e.g. ‘I do not have time to prepare vegetables; α = .730). Items were rated on a 5‐point Likert‐type scale ranging from 1 ‘Never true for me’ to 5 ‘Always true for me’. Participants then completed a sound check where they were required to identify the favourite colour given by the speaker as ‘Yellow’. Most participants correctly passed the sound check (i.e. 192 [97%]). Participants were then randomised to the control or intervention group.

##### Post‐intervention measures

Post‐baseline measures of habit, identity, intrinsic motivation and self‐efficacy were counterbalanced and administered before the behavioural measures of grocery shopping, vegetable consumption and demographics.

Habit was assessed using the four‐item Self‐reported Behavioural Automaticity Index (Gardner et al., 2012). Items were prefaced with the phrase: ‘When I am grocery shopping for dinner, buying vegetables is something …’. The four items included were (1) ‘I do automatically’, (2) ‘I do without having to consciously remember’, (3) ‘I do without thinking’ and (4) ‘I start before I realise, I'm doing it’. Items were rated on a 5‐point Likert‐type scale ranging from 1 ‘Strongly Disagree’ to 5 ‘Strongly Agree (*α* = .939).

Identity was assessed using an adapted version of the Exercise Identity Scale (Anderson & Cychosz, 1994). The scale incorporated four items: (1) ‘I consider myself someone who eats vegetables’, (2) ‘When I describe myself to others, I usually include my consumption of vegetables’, (3) ‘Others see me as someone who eats vegetables’ and (4) ‘Eating vegetables fits the way I want to live’. Items were rated on a 5‐point Likert‐type scale ranging from 1 ‘Strongly Disagree’ to 5 ‘Strongly Agree (α = .805).

Intrinsic motivation was measured for both vegetable shopping and consumption. These items were based on Phillips et al. (2019). The two items pertaining to shopping for vegetables were (1) ‘I enjoy shopping for vegetables in the supermarket or online’ and (2) ‘I think shopping for vegetables is rewarding’ (*α* = .822). The three items pertaining to eating vegetables were (1) ‘I enjoy eating vegetables’, (2) ‘I think vegetables are yummy’ and (3) ‘I think eating vegetables is rewarding’ (*α* = .869). Items were rated on a 5‐point Likert‐type scale ranging from 1 ‘Strongly Disagree’ to 5 ‘Strongly Agree’.

Self‐efficacy was assessed using two items created for the purpose of the present study: (1) ‘How confident are you that you will be able to purchase a variety (i.e., at least two types) of vegetables to eat with dinner in the next four weeks’ and (2) ‘How confident are you that over the next four weeks that you could overcome obstacles that prevent you from buying a variety of vegetables (i.e., at least two types) to eat with dinner? (α = 0.823). Items were rated on a 5‐point Likert‐type scale ranging from 1 ‘Very Unconfident’ to 5 ‘Very Confident’.

Grocery shopping for vegetables was assessed using three items created for the purpose of the present study. The first asked participants ‘On how many days did you go grocery shopping over the previous seven days?’. The second item asked participants ‘On how many of these days did you buy at least two types of vegetables to have with dinner’. Response options ranged from ‘0’ to ‘7’ for the first two items. The third item asked participants to list the different types of vegetables that they purchased over the previous 7 days to consume with dinner.

Vegetable consumption was measured in accordance with the pre‐screening procedure. Additionally, participants reported on their age, gender identity (i.e. ‘female’, ‘male’, ‘nonbinary’, ‘other’), household annual income in Great British Pounds and the number of household members who live off said annual income.

#### Follow‐up measures

At each follow‐up timepoint, participants reported on the aforementioned measures of barriers for buying and preparing/cooking vegetables, habit, identity, intrinsic motivation for both vegetable shopping and consumption and self‐efficacy (see Table 1 for reliability indices). These measures were counterbalanced and administered before the behavioural assessment of grocery shopping for vegetables. At each data collection point, participants reported the vegetables they purchased in an open‐ended response format and had the opportunity to submit up to three photographs of their shopping. Photographs were not submitted in the baseline survey.

**TABLE 1:** Cronbach's alpha scores for follow‐up measures.

	Week 1	Week 2	Week 3	Week 4	3 months	6 months
Barriers buying	.482	.588	.614	.683	.598	.582
Barriers cooking	.740	.811	.814	.832	.813	.813
Habit	.933	.943	.953	.955	.947	.944
Identity	.763	.788	.784	.803	.805	.794
Motivation shopping	.828	.873	.856	.875	.887	.833
Motivation eating	.855	.843	.870	.871	.842	.841
Self‐efficacy	.861	.924	.925	.926	.858	.901

### Intervention

Behaviour change techniques for each component are detailed in Table S1 (Michie et al., 2013). Participants in both groups were given the goal of increasing access to vegetables within their own home to ensure that they always had access to a variety of vegetables to eat with dinner. Participants in both groups were also given an educational video on how to prepare raw, steamed, boiled and roasted vegetables alongside links for vegetable recipes from jamieoliver.com (n.d.) and bbc.com (n.d.). These resources were chosen because they either specify the required skill level for the recipe (Oliver, n.d.) or they allow users to search for recipes by their favourite vegetable (BBC Food, n.d.). These resources can be viewed in full on OSF (https://osf.io/7zsx9/).

Participants in the intervention group watched a video detailing different examples (e.g. eating dinner with partner, eating dinner with children) of purchasing at least two types of vegetables to have with dinner and were further given an action‐ and coping‐planning intervention. In the action planning component, participants formed ‘if‐then’ plans for purchasing a variety of vegetables (e.g. If shopping for dinner ingredients, then I will purchase two vegetables to make with dinner). The coping planning component participants formed plans for how they would deal with possible barriers to executing their action plans (e.g. If there are no vegetables that I know I like at the store, then I will purchase two new vegetables to try with dinner). The intervention group was also given a mental imagery intervention based on Knäuper et al. (2011). Participants were told that imagining oneself being successful in executing a plan leads to greater success. They were asked to picture themselves grocery shopping and to imagine themselves picking up vegetables to buy for dinner. They were asked to imagine the look of the vegetables alongside their size, shape and colour. These resources can be viewed in full on OSF (https://osf.io/7zsx9/).

## STATISTICAL ANALYSES

### Power analysis

A power analysis for a linear multiple regression using a fixed model and *R* ^2^ increase (1 − *β* = 80%, *α* = .05) revealed that 159 participants were needed (i.e. 80 per group) for one tested predictor with three total predictors. The three predictor option was chosen as it is the largest number of predictors in any model and pertains to hypothesis 4. Means and standard deviations were taken from Phillips et al. (2019) and McGowan et al. (2013) to compute effect sizes between groups (Cohen's *d*). The smallest *d*‐value of .539 (*f* ^2^ = .073), from McGowan et al. (2013), was used. Because an interaction was tested, a conservative value of *f* ^*2*^ = .05 was used. It was preregistered that data would be collected from 200 participants (i.e. 100 per group) to allow for 20% attrition in the longitudinal surveys. Prolific reports that attrition on their longitudinal surveys is between 0% and 50% (n.d.).

### Coding of vegetables purchased

Participants self‐reported and submitted photographs of their vegetable shopping at the end of each follow‐up time point (weeks 1–4, months 3 and 6) to assess the number of different types of vegetables purchased. Vegetables were coded in accordance with the Britannica list of vegetables (Petruzzello, 2021) with the addition of mushrooms and bean sprouts which are known to count towards five servings of vegetables per day. Generic salad mixes were counted as one type of vegetable and were not counted when another specific type of leafy green was listed or photographed. Regarding packaged mixed vegetables, individual vegetables were coded when they were clearly identifiable in images or differentiated in text responses. When it was not clear, a conservative approach was taken as the bag was counted as a single type of vegetable or was not counted at all when other vegetables were listed. Due to the missing data for the photograph submissions across timepoints (e.g. 34.34% missing in Week 1) and low‐quality images in some cases, the self‐reported data was deemed to be more comprehensive and was utilised in the analysis to determine purchasing variety across follow‐up timepoints (i.e. H1b–H1c).

In accordance with preregistration, the data were cleaned using several steps. First, participants who failed at least two out of three random response checks were removed from analyses. Second, participants who did not complete the week 1 follow‐up were not eligible and their data was not utilised (Steegen et al., 2016). Third, participants in the intervention group who reported not completing either the planning or mental imagery components of the intervention were removed and the analyses were conducted (1) with intervention participants who completed at least some of the intervention components and (2) with intervention participants who completed all of the intervention components.

As preregistered, hypotheses 1a, 4 and 5a–b were tested using multi‐level modelling in SAS and hypotheses 1b–c and 2 were tested using independent samples *t*‐tests where the grouping variable was group assignment. Hypotheses 3a–c were assessed using multi‐level modelling in SAS, which was counter to the preregistered plan of using zero‐order correlations and linear regression analyses. However, this approach provides a more nuanced examination of the data. For all tested multi‐level models, week 1 was entered as the first time‐point. Time was coded as 1 (week 1), 2 (week 2), 3 (week 3), 4 (week 4), 12 (month 3) and 24 (month 6). Continuous level two variables were grand‐mean centred in the multi‐level models where relevant. Time‐varying covariates were grand mean centred specific to the intervention group in hypotheses 4 and 5. Associations between variables was assessed at the *p =* .05 level.

## RESULTS

### Preliminary results

A total of 380 participants completed the pre‐screening questionnaire with 98.7% (*N =* 375) indicating that they intended to do most of their own grocery shopping over the next 4 weeks. Additionally, 73% (*N =* 278) of participants reported consuming less than three servings of vegetables per day over the previous 7 days with 94% of these participants reporting that this was representative of their typical eating patterns. Of the 380 participants who completed the pre‐screening questionnaire, 272 were eligible for baseline.

A total of 203 participants completed both the baseline and week 1 surveys making them eligible for analysis. Specifically, only one participant from the control group who completed baseline did not complete the week 1 survey. As participants could complete the baseline anytime between survey administration and closure, eligibility was reanalysed concerning the quantity of vegetables consumed over the previous 7 days. Five participants (four intervention; one control) reported consuming more than three servings of vegetables per day over the previous 7 days. All five participants reported that this was indicative of their typical pattern of eating. Therefore, they were removed from analyses, which resulted in a total final sample size of 198 participants (103 control; 95 intervention). All participants passed both self‐reported random response checks, and 97% (*N =* 192) of participants passed the sound check. Thus, no participants met the preregistered threshold for exclusion based on random responding. All intervention participants completed both the action and coping planning components. Three intervention participants self‐reported not completing the mental imagery component of the intervention, which resulted in the analyses being conducted both with and without these participants. Main analyses are reported with the inclusion of all intervention (*N =* 95) participants when results did not differ, and any differences between analyses are reported (*N =* 92).

Attrition was low across all survey timepoints for eligible participants: Week 1 (*N =* 0; 0.00%), Week 2 (*N =* 7; 3.54%), Week 3 (*N =* 9; 4.55%), Week 4 (*N =* 9; 4.55%), 3 Months (*N =* 15; 7.58%) and 6 Months (*N =* 16; 8.08%). Given that multi‐level modelling can appropriately handle missing data, no participants were excluded due to these missing values. Most participants identified as female (54.5%; *N =* 107) or male (44.9%; *N =* 89), with one participant identifying as nonbinary (0.50%) and one participant choosing not to respond (0.50%). Participants ranged from 20 to 74 years of age (*Mx =* 41.03, *SD =* 11.08) and reported that including themselves 1 to 6 (*Mx =* 2.65, *SD =* 1.19) people lived off their household annual income (*Mx =* £44,283.17, *SD =* £41,203.24, *Minimum =* £0.00, *Maximum = £*500,000). On average, participants' household income was partitioned to £18,377.21 (*SD =* £14,255.86, *Minimum =* £0.00, *Maximum =* £125,000) for each dependent member of their household including themselves. Generally, there were no significant differences between the intervention and control group in terms of the number of days that they went grocery shopping or the number of days that they purchased vegetables across the follow‐up timepoints (*ps* = .099 to .919). This is except for Week 4 where participants did not differ on the number of days that they went grocery shopping (*p =* .099) but did differ on the number of days that they bought vegetables (*p =* .026). Here, The control group participants bought vegetables an average of 1.11 days (*SD =* .75) and the intervention group participants bought vegetables on an average of 1.38 days (*SD =* .88).

### H1: The impact of planning on habit development

Hypothesis 1a, that participants given an action‐ and coping‐planning intervention would form stronger higher order habits for vegetable shopping in comparison with the control group over the 4‐week period, and 3‐ and 6‐month follow‐ups, was tested using multi‐level modelling with linear, quadradic and cubic measures of time. Results showed higher order habit formation occurred for both groups over time and that this increase was cubic, with participants in the intervention group having stronger higher order habits than those in the control group at the final 6‐month follow‐up (Table 2).

**TABLE 2:** Hypothesis 1a: The impact of planning on habit development, multi‐level modelling results.

	*Estimate*	*SE*	*df*	*t*	*p*
Results without baseline habit strength					
Intercept	3.575	0.286	196	12.51	<.001
Time	−0.261	0.091	928	−2.88	.004
Time * time	0.031	0.010	928	3.19	.002
Time * time * time	−0.001	<0.001	928	−3.27	.001
Group	−0.352	0.183	196	−1.92	.056
Group * time	0.180	0.058	928	3.10	.002
Group * time * time	−0.018	0.006	928	−2.93	.002
Group * time * time * time	0.001	<0.001	928	2.89	.004
Results with mean‐centred baseline habit strength as a covariate					
Intercept	3.356	0.222	195	15.17	<.001
Baseline habit	0.686	0.044	195	15.44	<.001
Time	−0.260	0.091	928	−2.88	.004
Time * time	0.031	0.009	928	3.18	.002
Time * time * time	<−0.001	<0.001	928	−3.27	.001
Group	−0.207	0.142	195	−1.46	.147
Group * time	0.179	0.058	928	3.09	.002
Group * time * time	−0.018	0.006	928	−2.92	.004
Group * time * time * time	<0.001	<0.001	928	2.87	.004
Results with mean‐centred baseline habit strength as a moderator					
Intercept	3.306	0.219	194	15.09	<.001
Baseline habit	0.917	0.200	194	4.58	<.001
Time	−0.243	0.090	922	−2.70	.007
Time * time	0.030	0.009	922	3.10	.002
Time * time * time	<−0.001	<0.001	922	−3.21	.001
Group	−0.172	0.140	194	−1.22	.222
Group * time	0.164	0.058	922	2.83	.005
Group * time * time	−0.017	0.006	922	−2.76	.006
Group * time * time * time	<.001	<.001	922	2.74	.006
BH * time	0.096	0.082	922	1.16	.247
BH * time * time	−0.013	0.009	922	−1.46	.144
BH * time * time * time	<0.001	<0.001	922	1.52	.129
Group * BH	−0.043	0.124	194	−0.34	.732
Group * BH * time	−0.116	0.051	922	−2.28	.023
Group * BH * time * time	0.012	0.006	922	2.27	.024
Group * BH * time * time * time	<−0.001	<0.001	922	−2.22	.026

*Note*: Baseline Habit = BH. Outcome is the Self‐reported Automaticity Index.

**FIGURE 3:**
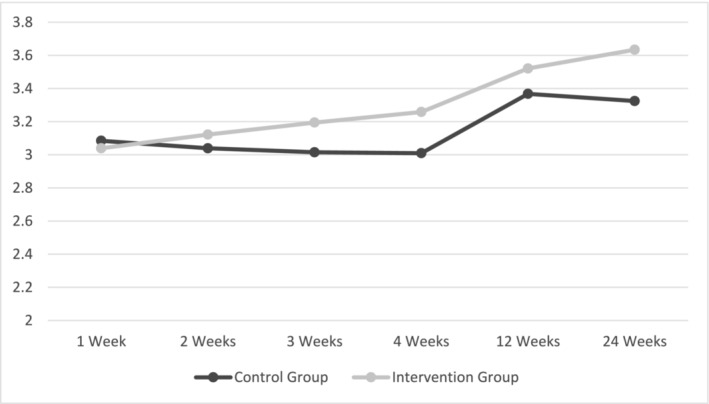
H1a results with mean‐centred baseline habit strength as a covariate. *Note*: Outcome is the Self‐reported Automaticity Index.

**FIGURE 4:**
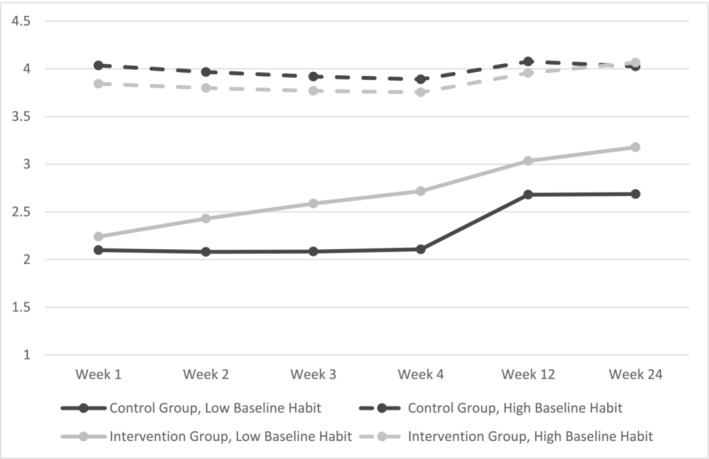
H1a results with mean‐centred baseline habit strength as a moderator. *Note*: Outcome is the Self‐reported Automaticity Index.

As there were a range in habit scores at baseline, in an exploratory analysis, baseline habit strength was entered, first as a covariate and second as a moderator, in a multi‐level model to determine if the intervention was more effective for intervention participants versus control participants who did not have a habit at intake. Results utilising baseline habit as a covariate replicated the main results insofar that there was a cubic interaction between time and group with the intervention group having stronger habits than the control group at the 6‐month follow‐up (Table 2; Figure 3). Results using baseline habit as a moderator showed that the intervention was more effective than the control condition for participants who had low levels of baseline habits (Table 2; Figure 4).

Hypothesis 1b, that participants in both the intervention and control group would purchase two or more types of vegetables per week, was tested using a series of *t*‐tests. This hypothesis was supported, and there was no significant difference between the two groups at any of the timepoints (*ps* = .076–.691), including baseline, with both groups purchasing an average of at least two types of vegetables at each measurement. Across *t*‐tests Cohen's *d* effect sizes were <.10, indicating a trivial effect and further supporting that there were no between‐group differences.

Hypothesis 1c, that participants in both the intervention and control group would both purchase a variety of vegetables across weeks (i.e. more than two types of vegetables) and that this difference would be nonsignificant, was tested using an independent samples *t*‐test. As predicted, there was no significant difference between the two groups on the variety of vegetables that they purchased across timepoints (*p =* .376) with the control group purchasing an average of 10.74 (*SD* = 3.96) types of vegetables and the intervention group purchasing an average of 10.93 (*SD* = 4.41) types of vegetables across the study timepoints including baseline. The Cohen's *d* effect size was −0.05, indicating a trivial effect which supports our hypothesis of there being no between group differences.

### H2: The immediate impact of planning on mechanisms of action

Hypothesis 2, that participants who were given an action‐ and coping‐planning intervention would have higher self‐efficacy, in comparison with the control group, immediately post‐intervention and that there would be no differences between the two groups on healthy‐eater identity, habit or intrinsic motivation immediately post intervention, was tested using independent samples *t*‐tests. This hypothesis was only partially supported. Specifically, although there were no between‐group differences on identity, habit or intrinsic motivations, the control group had higher self‐efficacy immediately post‐intervention compared with the intervention group (see Table 3). This difference was small in magnitude and did not persist across the follow‐up time points (hypothesis 3). When the three intervention participants who did not complete the mental imagery intervention were removed, there were no significant differences on self‐efficacy (*p* = .069), or any other proposed mechanisms of action, immediately post intervention.

**TABLE 3:** Hypothesis 2: The immediate impact of planning on the mechanisms of action, independent *t*‐test results.

	*t*	*df*	*p*	Mean difference	*SE*	Cohen's *d*
Self‐efficacy	1.676	196	.048	0.214	0.127	0.238
Habit	1.308	196	.096	0.211	0.162	0.186
Identity	0.344	196	.366	0.0426	0.124	0.049
Motivation shopping	−0.800	196	.212	−0.113	0.141	−0.114
Motivation eating	0.054	196	.479	0.007	0.126	0.008

*Note*: The grouping variable is intervention versus control group.

### H3: The longer term impact of planning on the mechanisms of action

Hypothesis 3a, that self‐efficacy, intrinsic motivation for consuming, and shopping for vegetables, and healthy‐eater identity (i.e. mechanisms of action) would increase more strongly over time for the intervention group in comparison with the control group, was tested using multi‐level modelling for each outcome across both groups. None of the proposed mechanisms of action varied between groups or over time (i.e. linear, quadradic, cubic; *p* > .05). Moreover, there were no significant interactions between time (i.e. linear, quadradic, cubic) and group (*p* > .05).

Hypothesis 3b, that the mechanisms of action would predict the frequency of grocery shopping trips where participants purchased at least two types of vegetables, was assessed with multi‐level modelling that included all mechanisms as predictor variables across both groups. Neither the proposed mechanisms of action nor time (i.e. linear, quadradic, cubic; *p* > .05) predicted the number of days participants purchased at least two types of vegetables. Additionally, there were no significant interactions between time (i.e. linear, quadradic, cubic) and group (*p* > .05).

Hypothesis 3c, that the mechanisms of action would predict habit formation, was tested with multi‐level modelling that included all mechanisms as predictor variables across both groups. The main effect of identity predicted habit formation (*t =* 4.47, *p <* .001). However, the other proposed mechanisms, as well as time (i.e. linear, quadradic, cubic), were not predictors of habit formation, nor were there any significant interactions between time (i.e. linear, quadradic, cubic) and group (*ps* > .05).

### H4: Frequency of grocery shopping trips as a moderator of habit development

**FIGURE 5:**
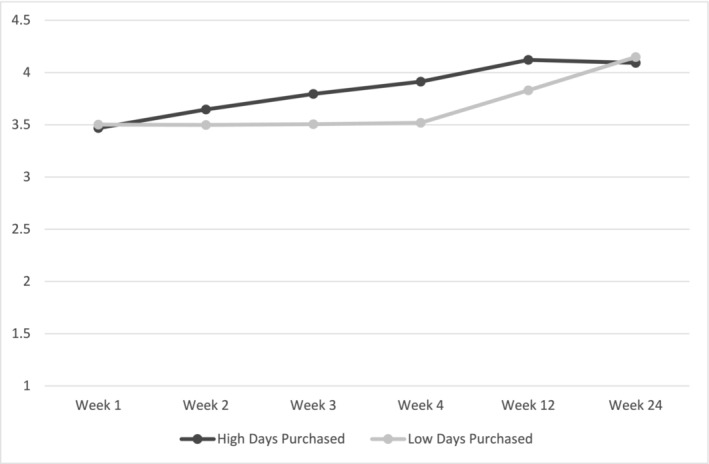
H4 results while controlling for mean‐centred baseline habit strength. *Note*: outcome is the Self‐reported Automaticity Index.

Hypothesis 4, that those who engaged in more grocery shopping trips where vegetables were purchased, post‐intervention, within the intervention group would have a higher level of habit formation for shopping for vegetables, was tested using multi‐level modelling while controlling for baseline habit strength and baseline purchasing days. The cubic interaction term was significant, and results showed that within the intervention group, participants who purchased vegetables more frequently formed stronger habits at Weeks 1–12, but that this effect dissipated by the 6‐month follow‐up (Table 4; Figure 5). Results were consistent when baseline habit strength was not controlled for.

**TABLE 4:** Hypothesis 4: Frequency of grocery shopping as a moderator of habit development, multi‐level modelling results.

	*Estimate*	*SE*	*df*	*t*	*p*
Intercept	3.054	0.108	93	28.04	<.001
Mean‐centred baseline habit	0.575	0.062	93	9.23	<.001
Mean‐centred days purchased	−0.138	0.087	434	−1.59	.114
Time	0.095	0.045	434	2.11	.036
Time * time	−0.005	0.005	434	−1.08	.279
Time * time * time	<0.001	<0.001	434	0.81	.418
Days purchased * time	0.133	0.049	434	2.74	.007
Days purchased * time * time	−0.012	0.005	434	−2.37	.018
Days purchased * time * time * time	<0.001	<0.001	434	2.09	.037

*Note*: Outcome is the Self‐reported Automaticity Index.

### H5: Barriers to purchasing and preparing vegetables as a moderator of habit development

**FIGURE 6:**
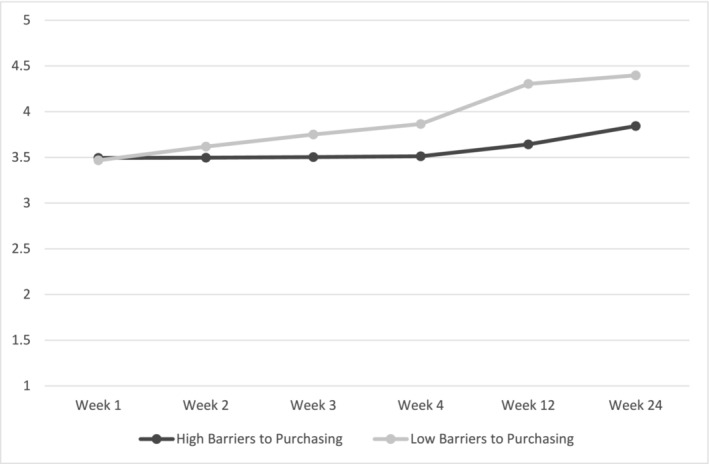
H5a results while controlling for mean‐centred baseline habit strength. *Note*: outcome is the Self‐reported Automaticity Index.

Hypothesis 5a, that participants who perceived more barriers to purchasing vegetables within the intervention group would have lower levels of habit formation for shopping for vegetables, was tested using multi‐level modelling with baseline habit and baseline barriers to buying as control variables. The hypothesis was marginally supported insofar that participants in the intervention group who perceived more barriers to purchasing vegetables had lower levels of habit formation across the intervention follow‐ups (*p =* .053; Table 5; Figure 6). This linear interaction was marginally significant and participants who perceived fewer barriers to shopping for vegetables formed stronger habits across the follow‐up timepoints. Results were consistent when not controlling for baseline habit strength. When the three intervention participants who did not complete the mental imagery intervention were removed, the linear interaction term was significant (*p =* .010).

**TABLE 5:** Hypothesis 5a and 5b: Barriers to purchasing and preparing vegetables as a moderator of habit development, multi‐level modelling results.

	*Estimate*	*SE*	*df*	*t*	*p*
Hypothesis 5a					
Intercept	3.071	0.110	93	27.85	<.001
Mean‐centred baseline habit	0.570	0.0633	93	9.01	<.001
Mean‐centred barriers to purchasing	0.144	0.154	434	0.93	.351
Time	0.090	0.045	434	2.01	.045
Time * time	−0.004	0.005	434	−0.93	.353
Time * time * time	<0.001	<0.001	434	0.63	.528
Barriers * time	−0.134	0.070	434	−1.94	.053
Barriers * time * time	0.009	0.007	434	1.22	.222
Barriers * time * time * time	<−0.001	<0.001	434	−0.91	.363
Hypothesis 5b					
Intercept	3.124	0.107	93	29.35	<.001
Mean‐centred baseline habit	0.478	0.059	93	8.12	<.001
Mean‐centred barriers to cooking	−0.322	0.114	434	−2.82	.005
Time	0.062	0.044	434	1.41	.159
Time * time	−0.003	0.005	434	−0.60	.548
Time * time * time	<0.001	<0.001	434	0.42	.675
Barriers * time	−0.058	0.055	434	−1.06	.289
Barriers * time * time	0.004	0.006	434	0.76	.450
Barriers * time * time * time	<−0.001	<0.001	434	−0.60	.546

*Note*: Outcome is the Self‐reported Automaticity Index.

Hypothesis 5b, that participants who perceived more barriers to preparing vegetables within the intervention group would have lower levels of habit formation for vegetable shopping, was tested using multi‐level modelling with baseline habit and baseline barriers to cooking as control variables. Results were supported insofar that barriers to preparing and cooking vegetables was negatively related to habit formation (Table 5). This relationship did not vary over time, and participants in the intervention group who perceived fewer barriers had stronger habit formation. Results were consistent when not controlling for baseline habit strength.

## DISCUSSION

Extending on previous research (i.e. Kilb & Labudek, 2022; Phillips et al., 2019), the present study contributes to the new and growing literature on higher order habit formation by taking a novel approach and demonstrating the real‐world utility of a higher order habit intervention for the initial step of most nutrition behaviours—shopping. Specifically, this study tested an action‐ and coping‐planning alongside mental imagery intervention on the formation of higher order habits for vegetable shopping over a 4‐week period as well as at 3‐ and 6‐month follow‐ups.

Results indicated that the intervention group had stronger higher order habit formation than the control group at the 6‐month follow‐up and that the intervention was particularly effective for participants with low baseline higher order habits for vegetable shopping. These findings extend those from Phillips et al. (2019) and Kilb and Labudek (2022) by demonstrating that a simple, one‐off, intervention can lead to long‐lasting change in higher order habits for complex behaviours within the nutrition domain. Importantly, higher order habit formation was achieved, in comparison with lower order habit formation, as participants bought a variety of vegetables within and across each time point. Although habit formation seemed to plateau in the control group at the 6‐month follow‐up, habit formation was still on an upward trajectory for the intervention group. This is important as identifying effective intervention strategies to promote a sustained well‐balanced diet, such as by improving vegetable access within the home environment, is a critical first step for improving public health outcomes and reducing the prevalence of diet‐related chronic illness (e.g. Almeida‐de‐Souza et al., 2018; Büchner et al., 2010; Cooper et al., 2012; Meengs et al., 2012).

As hypothesised, the intervention did not have an immediate effect on identity or intrinsic motivation for vegetable shopping or eating vegetables. However, counter to expectations, the control group had higher self‐efficacy immediately post intervention, possibly as a result of the control condition being less complex and not having to reflect on barriers to success (i.e. coping planning). Importantly, this result was not robust as this effect was null when participants who did not complete the entirety of the intervention were removed from the analysis.

Intrinsic motivations and self‐efficacy did not differ between groups across the remainder of the study nor did they predict habit formation or the frequency of shopping for vegetables. Identity did not vary between groups at baseline or over the course of the intervention nor did it predict shopping behaviour. However, identity was related to habit formation, which is in line with the Multi‐Process Action Control Framework and previous research (e.g. Rhodes, 2021). However, the relationship between health‐related habit and identity formation and how they interact to co‐determine behaviour is not well understood and more research is needed into this area. Although theoretical frameworks often propose that intrinsic motivation and self‐efficacy can play a role in habit formation (e.g. Gardner & Lally, 2013; Wood & Rünger, 2016), the current findings challenge this view and emphasise the need to further explore what the mechanisms of habit formation are and how to target these for routine behaviours, such as grocery shopping. Additionally, it is important to acknowledge that this study did not assess all potential mechanisms involved in translating initial intentions into habitual behaviour (e.g. action control, intrinsic rewards; Orbell & Verplanken, 2018; Phillips et al., 2016), further emphasising the need for more research in this area.

Although more frequent grocery shopping trips in which vegetables were purchased initially strengthened habits within the intervention group, this effect diminished at the 6‐month follow‐up. Thus, as expected, habit formation was quicker for those who had more opportunities to practice pairing their chosen cue with behaviour. Finally, barriers to vegetable shopping and preparing vegetables was negatively associated with habit formation in the intervention group, with stronger higher order habits being formed in those who had fewer barriers to their success. This highlights the need for interventions to address not only individual level factors, such as education on how to prepare nutrient dense foods (Pem & Jeewon, 2015), but also the need for society more broadly to address structural barriers such as the price and accessibility of produce (e.g. food deserts; Karpyn et al., 2019).

Although this study contributes a novel test of higher order habits in the domain of nutrition that extends upon previous studies by acknowledging the complexity of eating behaviours and using a long‐term follow‐up, the present study is not without limitations. First, although all participants did not meet the daily recommended serving of three portions of vegetables, there was a subgroup of participants who reported already having a higher order habit for buying two vegetables to consume with dinner at baseline. This indicates the need for supplemental strategies such as targeting vegetable purchasing and consumption at other meal times (i.e. breakfast, lunch and snacks) to promote daily intake compliance. Second, the measure of barriers to buying vegetables had low reliability throughout baseline and the follow‐up timepoints (see Table 1). This is likely a formative construct—with another example of this type of construct being socioeconomic status—whereby items cause the latent construct rather than a more traditionally seen reflective measure whereby the latent construct causes the items (Freeze & Raschke, 2007). For formative constructs, correlations between items are not expected to be high. However, more methodological work will need to be done to validate this measure as formative. Third, the current sample also consisted of participants in households that were, on average, living above the median household income. Specifically, participants reported an average household income of £44,283.17, which is considerably higher than the median household income at the time of £35,000 (Office for National Statistics, 2023). Therefore, modifications may be needed to promote an increase in vegetable intake among lower socioeconomic households who may experience additional barriers to accessing vegetables.

Empirical evidence from this study, along with findings from prior research (Kilb & Labudek, 2022; Phillips et al., 2019), suggests that higher order habits, like lower order habits, can be developed through behavioural repetition. This evidence primarily emerges from studies conducted in familiar contexts, such as university dining halls, home environments and physical or virtual supermarkets. While these contexts are important and likely make up most individuals daily routines, a critical theoretical question remains: How can higher order habit formation and maintenance be optimally supported in novel contexts? Examples of such contexts include selecting or ordering vegetables while traveling abroad or establishing gym attendance routines among non‐exercisers. Higher order habits may offer distinct advantages in novel settings as they provide greater flexibility in execution. For instance, higher order habits could allow individuals to adapt when preferred vegetables are unavailable or when gym equipment is occupied or under repair. However, a certain level of base knowledge may be required to reap this advantage.

### CONCLUSION

Nutrition is a cornerstone of good health as a well‐balanced diet is a known protective factor against several common ailments (e.g. heart disease, stroke; Wang et al., 2021; World Health Organization, 2019). Higher order habit interventions are one avenue that may help increase the likelihood that individuals initiate and maintain such health behaviours (Phillips et al., 2019). Using a randomised controlled trial, the current study found that participants who were administered a simple planning intervention alongside mental imagery for shopping for vegetables formed stronger higher order habits than participants who were only given a behavioural goal alongside intervention on food preparation. Although further research is needed, findings from the current study highlight that higher order habit interventions have the potential to support the maintenance of complex health behaviours. Importantly, the tested intervention can be administered remotely and is both time‐efficient for participants and cost‐effective to administer, making it a scalable approach.

## CONFLICT OF INTEREST STATEMENT

The authors declare no conflicts of interest.

## ETHICS STATEMENT

Ethical approval was obtained from the University of Dundee prior to beginning data collection (UoD‐SoSS‐PSY‐STAFF‐2022‐109). Additionally, informed consent was sought prior to collecting data from individual participants (p. 7).

## Supporting information


**Table S1.** Behaviour Change Strategies Per Intervention Group

## Data Availability

Except for photographs and open‐ended response which may contain identifiable data, the quantitative data are openly available and archived on the Open Science Framework (https://osf.io/7zsx9/?view_only=07978b071059431697a73a054959a319).
