# Supplementary material for: Rooted in routine: Fostering higher order vegetable‐shopping habits using a randomised simple planning intervention
Source: Appl Psychol Health Well Being. 2025 Jan 30;17(1):e12649. doi: 10.1111/aphw.12649 (PMC11782107; doi:10.1111/aphw.12649)
Supplement: Supplementary file 1 — Table S1. Behaviour Change Strategies Per Intervention Group [file APHW-17-0-s001.docx]

**Supplementary Table 1**

*Behaviour Change Strategies Per Intervention Group*

|  | Control  Group | Identity  Group |
| --- | --- | --- |
| Habit Formation/Action Planning (8.3; 1.4) |  | **✓** |
| Behavioral Practice/Rehearsal (8.1) |  | **✓** |
| Problem Solving (1.2) |  | **✓** |
| Mental Rehearsal of Successful Performance (15.2) |  | **✓** |
| Credible Source (9.1) | **✓** | **✓** |
| Self-monitoring of Behaviour (2.3) | **✓** | **✓** |
| Goal Setting (1.1) | **✓** | **✓** |
